# Community-Wide Distribution of a Catalytic Device to Reduce Winter Ambient Fine Particulate Matter from Residential Wood Combustion: A Field Study

**DOI:** 10.1371/journal.pone.0166677

**Published:** 2016-11-30

**Authors:** Olivia Johnston, Fay Johnston, John Todd, Grant Williamson

**Affiliations:** 1 School of Health Sciences, University of Tasmania, Launceston, Tasmania 7250, Australia; 2 Menzies Institute for Medical Research, University of Tasmania, Private Bag 23, Hobart 7000, Tasmania, Australia; 3 School of Natural Sciences, Edith Cowan University, Joondalup, Western Australia 6027, Australia; 4 School of Biological Sciences, University of Tasmania, Private Bag 55, Hobart 7001, Tasmania, Australia; University of the Chinese Academy of Sciences, CHINA

## Abstract

Residential wood combustion is the main source of elevated concentrations of fine particulate matter (PM_2.5_) during winter in many towns of Tasmania, Australia. A commercially available firebox catalyst in Australia has previously been shown to reduce visible smoke emissions and the manufacturer reports reductions in particle emissions generated from individual wood heaters in laboratory settings. This study aimed to evaluate the potential for community-wide distribution of the catalyst to improve the ambient winter air quality in the field. The study was set in four rural towns in northern Tasmania with similar topography, population size, and proportion of houses using wood heaters for space heating. Hourly PM_2.5_ concentrations and meteorological conditions were monitored in all locations by fixed stations from May-September, 2013 and 2014. In June 2014, residents of one town, Perth, were offered a free catalyst for placement in their fireboxes. A general linear model evaluated the impact of the intervention using an indicator variable adjusted for hourly conditions of weather. Almost 80% of wood heater owners in Perth accepted a catalytic device. However, no significant changes in ambient PM_2.5_ concentrations were associated with the catalyst trial. Future community-level research should address maintenance of the catalyst in the firebox, and the adequacy of conditions that facilitate catalysed combustion in individual heaters.

## Introduction

Residential wood smoke is a significant source of winter air pollution in many parts of the world and is associated with important harmful public health impacts. For example, direct associations between wood smoke and population mortality have been reported in Launceston Tasmania [1], and with hospital admissions for cardiovascular and respiratory diseases in Christchurch New Zealand [2] and Temuco Chile [3].

**Fig 1:**
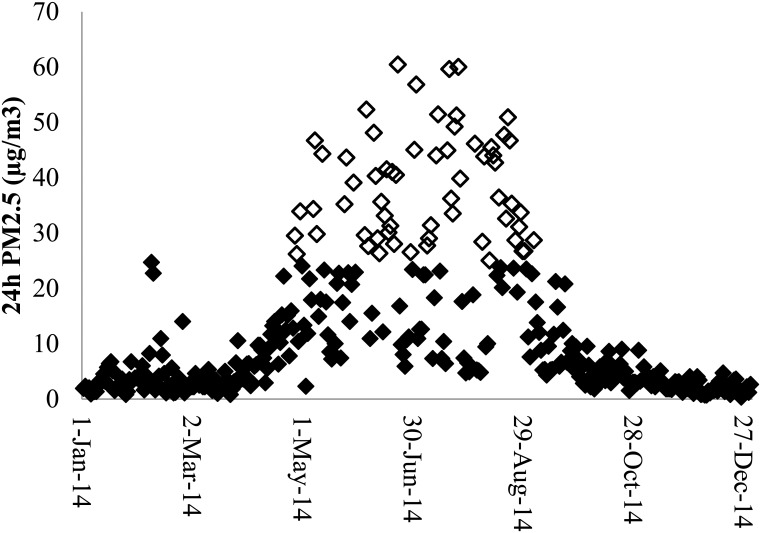
24 hour average PM_2.5_ in Longford Tasmania in 2014. Winter wood smoke dominates from May to August. Some incidents of bushfire smoke occurred in February. Hollow diamonds indicate PM_2.5_ > 25μg/m^3^.

Efforts to reduce residential wood smoke require ambient air quality monitoring to identify communities experiencing high ambient PM_2.5_ concentrations and to evaluate changes over time. In the Australian state of Tasmania, the development of the ‘Base Line Air Network of the EPA Tasmania’ (BLANkET) provides continuous, real-time measurement of PM_10_, PM_2.5_ and meteorological parameters at 29 locations including cities, towns and rural locations [4]. This network has shown that winter wood smoke is a problem in many smaller towns as well as larger cities. For example, the town of Longford (population about 3,000) experienced 61 days in 2014 where 24 hour PM_2.5_ exceeded the Australian standard for ambient air 24 hour particle concentration for particles as PM_2.5_ of 25μg/m^3^ (Fig 1).

Pollution control authorities are attempting to reduce the emissions of smoke through education and other intervention approaches.

Environmental and health authorities recognize the need to reduce this source of air pollution. One intervention known to achieve good results is to ban the use of firewood for space heating. Significant reduction in the number of households using firewood is also successful. For example, in Launceston, Tasmania, the number of wood heaters was reduced by almost 50% through a two year, $2 million program of advertisements emphasising the health impacts of wood smoke, smoke patrols identifying particularly smoky chimneys, subsidies to remove wood heaters and the introduction of a special lower electricity tariff for use with certain electrical heaters [1]. However, if affordable space heating fuels are not available then the adverse health impacts of cold homes make authorities reluctant limit the options for domestic heating.

Other intervention approaches have not proven successful in Tasmania. They include the gradual introduction of lower emission standards for wood heaters sold in Australia. This has not proved successful due to slow adoption and widespread non-compliance with national emission standards [5]. However, a rapid changeover in Libby, Montana, in which conventional wood heaters were replaced with new low emission models, reduced ambient PM_2.5_ concentration by 30% [6]. The Tasmanian Government introduced legislation allowing pollution control authorities to fine households emitting excessive visible smoke [7]; but reluctance by local authorities to take homeowners to court has meant no fines have been issued. Community education campaigns aimed at improving the operation of heaters have also been unsuccessful in reducing ambient fine particle concentrations [4,8].

One intervention approach that was reported as successful took place in Armidale, NSW Australia where a commercially available catalytic device was placed in the firebox of study participants, and chimney emissions were compared with no intervention, and with educational interventions [9]. The study used two independent observers, blinded to the status of the household with respect to their intervention status, to visually assess the density of chimney smoke plumes according to a standard protocol. The assessments were conducted on six separate occasions before and after the intervention. This research concluded that use of the catalyst was associated with significantly reduced smoke emissions from individual chimneys but there was no quantification of changes in ambient particle concentrations.

**Fig 2:**
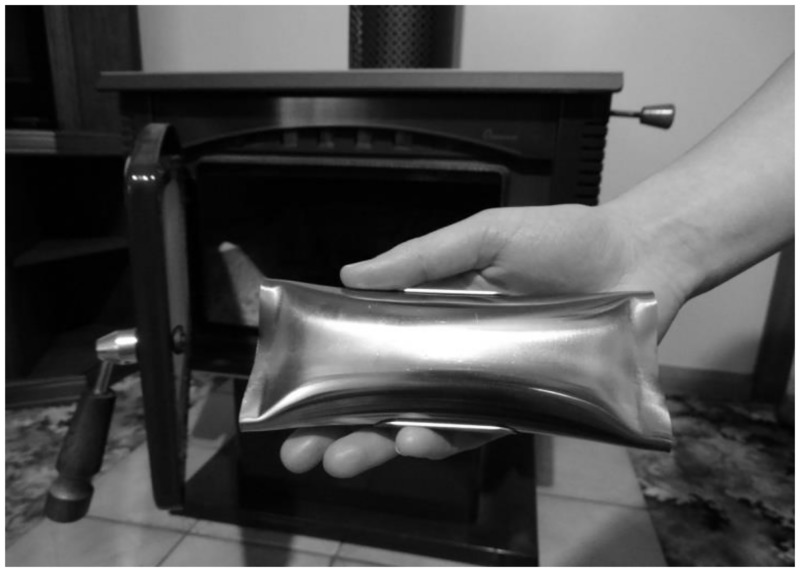
Catalytic combustor (15cm long) used in this program.

If effective, small catalytic combustors could potentially provide a practical and economical approach to reducing residential wood smoke emissions. Wood combustion catalysts have been reported to reduce emissions of PM and PAH by up to 30% via thermal oxidation [10,11]. Simple installation of a catalytic device into the combustion chamber of a wood heater, without altering wood heater operation, may be more acceptable and achievable by most members of a community. The small, commercially available catalytic device, *SmartBurn*^®^ (Fig 2), used in the Armidale study is promoted in Australia to reduce accumulation of soot and creosote within the flue and firebox. However its precise mechanism of action, other than being described as a catalyst, has not been made public. The steel tubular device encases a solid catalytic mixture which has been tested and approved for sale by the relevant Australian authorities. Although the specific components in the catalytic device have not been made public, the mechanism of action is described on the company website as follows [12]. As combustion temperature rises, a fraction of the mixture vaporises, escaping from open ends of the casing improving combustion through catalytic ignition of combustible gases within the firebox. The catalyst is reported to have an average lifetime of three months. Within the firebox, the canister must be placed level and to one side and remain level and elevated, maintaining the openings free for vapour escape and subsequent oxidation of combustible compounds. The catalyst has been reported to reduce particulate emissions from a single heater by up to 54% in laboratory tests. These results are available via the manufacturer’s website, but no similar studies are currently available in the academic peer-reviewed literature [13]. The field trial described above identified an association with a reduction in the density of visible smoke plumes from wood heaters [9]. However, it remains the only available peer reviewed study of the product. Effectiveness in studies of individual heaters does not automatically translate into a practical community wide intervention and the influence of this technology upon ambient pollution has not been previously quantified. Independent peer reviewed research is essential to guide public health policy. If similar results are achieved in community settings, there is the potential for this to provide a practical method to reduce the burden of ill health associated with poor winter air quality.

The present study was set in Northern Tasmania, incorporating the rural towns, Perth, Longford, Hadspen and Westbury, with respective populations of 2567, 3053, 2063 and 1476 [14]. As with most rural towns of Tasmania, wood combustion is the main type of space heating, with approximately 50% of households operating a wood heater [4]. Daily mean ambient PM_2.5_ mass concentrations often exceed 25μg/m3, especially on winter nights with low temperature and low wind speed [4]. The four towns are all located on catchments of the Tamar River within 50km of each-other and have very similar topography, being in the same valley system. The climate of all four towns is cool-temperate, with warm, dry summers and cool, wet winters. Mean summer (January) maximum temperatures in the region are 25°C while winter (July) minimum temperatures are 2°C, and annual rainfall is approximately 600mm. The towns are situated in an agricultural area, with a mix of dry-land and irrigated cropping and pasture, with minimal traffic or other sources of particulate pollution during the winter. There are no significant industrial pollution sources. The median age of the population in the region is 43, with the most common occupations being technical and trade workers, managers and labourers.

This paper presents an evaluation of the influence of community- wide distribution of a catalytic device on Perth’s winter ambient air quality. It was hypothesised that the post-intervention concentration of ambient PM_2.5_ would be lower than pre-intervention in the intervention town but not the three comparison towns.

## Methods

### Study design

We conducted a before and after intervention trial in Perth, and compared the results with the nearby towns of Longford, Hadspen and Westbury, that have similar air quality. The study followed a Before-After-Control-Impact (BACI) design, including two time periods with three control sites and a single intervention site. BACI designs are commonly used in environmental field studies and are particularly appropriate when a possible step change, rather than a gradual change in environmental conditions is being assessed [15]. Although two of the three comparison towns had received some education promotions in the years of 2013 (Hadspen) and 2014 (Longford), subsequent evaluation demonstrated that these campaigns had no influence on air quality or wood burning practices in these towns [8].

The main outcome measure was the change in hourly average particulate matter concentrations between pre- and post- intervention phases.

The Tasmania Social Sciences Human Research Ethics Committee approved the research presented in this paper.

### Fixed ambient air sampling

The BLANkET air monitoring stations of Perth, Longford, Hadspen and Westbury were used to measure hourly mean PM_2.5_ concentrations and meteorological parameters. PM mass concentration was measured at 10-minute intervals via a calibrated non-gravimetric, low-volume, light scattering optical particle counter (OPC), the 8533 DRX DustTrak [16]. DustTrak field instruments are installed in specially designed weatherproof enclosures with sampling inlets heated to 40°C. The heaters were developed and installed within each device to counteract the influence of weather conditions, such as humidity and fog, upon particulate sampling. Reference DustTrak instruments in continual operation at two separate locations in Tasmania are calibrated against accredited reference low-volume gravimetric air samplers (TEOM 1400AB) with ambient particle size distributions (i.e. wood smoke dominant) similar to those in the target locations. All DustTrak monitors used in this study are regularly calibrated against one of the two reference devices using a ‘smoke box’ with variable wood smoke concentrations [16]. The DustTrak samplers were located at secure sites near the geographic centre of the urbanized areas of each of the targeted towns. Wireless Davis Vantage Pro 2 weather stations at each site measured meteorological parameters of wind speed, atmospheric pressure, ambient temperature and relative humidity via an anemometer, a barometer and temperature and humidity sensors. Ambient air quality data of the four locations from 2013 and 2014 were analysed for the colder months only (i.e. excluding the seven months of October through to April). Data were allocated to one of two sampling phases as follows: pre-intervention (May 17, 2013 to June 14, 2014) and post-intervention (July 1, 2014 to September 30, 2014).

### The intervention

Community engagement activities were conducted in the months leading up to the intervention to inform residents of the planned intervention. These included advertisements in the local newspaper, poster displays, presentations to local groups including the Lions Club and local government council.

On the weekend of the 14–15 June 2014, all households identified as using firewood for domestic space heating were offered a free catalyst. Canisters were donated by Smartburn Australia. The company provided education to the research team and volunteers in the correct techniques for inserting and maintaining the product but was not otherwise involved in the design implementation, evaluation and reporting of results. Volunteers, consisting of students and staff of the University of Tasmania, visited every house and recorded information including the residential address, presence of a wood heater (indicated by a heater flue), whether or not residents were home and willing to participate, and the number of canisters delivered per household. The volunteers offered to install a catalytic device in all wood heaters on their property. Residents were told how to install and maintain the catalyst. Specific education about optimal operation of their heater was not a part of the intervention, but is included in the packaging of each individual product. Residents who were not at home during the weekend of door-knocking were left a flyer, which could be exchanged for a free catalyst at a local service station.

### Participant survey

Three months after the distribution of the catalysts residents of the intervention community of Perth were surveyed by email and telephone to identify observed impacts of the catalyst among participants and any factors that may influence function of the device. Respondents reported the type and age of their wood combustion appliance, and typical durations that complete primary combustion airflow is maintained after ignition and reloading. Wood moisture was indicated by self-reported durations and conditions of firewood storage. Respondents also reported the duration and conditions of placement and maintenance of the canister within the firebox. Any changes to the wood heater observed throughout canister use were reported and intentions to purchase the catalyst in the future were indicated. Each survey lasted approximately 5–10 minutes.

### Statistical analyses

Statistical analyses were conducted in two stages. First, we adjusted for the variation in daily PM_2.5_ concentrations attributable to the meteorological conditions using a RandomForest model [17]. A RandomForest model constructs a series of regression trees based on subsets of input variables and data, and generates predictions by combining the output of the regression trees, weighted by the strength of their fit. This model is capable of incorporating non-linear associations between predictor and response variables, allowing the influence of meteorological variables on PM_2.5_ to be controlled for more accurately than in a linear model. The RandomForest model used PM_2.5_ as the response variable and the predictor variables included wind speed, relative humidity, atmospheric pressure, temperature, hour of day, and day of week. Residuals generated by this model, representing variation in PM_2.5_ after these meteorological variables have been taken into account, were then used in the second stage of modelling to test for a change associated with the intervention.

For this we used a generalised linear model (GLM) to correlate the residual mean hourly PM_2.5_ concentrations with time period and location as interacting predictor variables to identify an interaction between location and intervention phase. Such an interaction would determine the amount of variation in PM concentration that may be attributed to the intervention after adjustment for meteorological parameters. The analyses were repeated for the specific hours of 7.00am, 6.00pm and 11.00pm, when ambient smoke concentrations were highest throughout the day. All analyses were conducted using R version 3.1.0. Results from the survey were summarised using descriptive statistics.

## Results

Perth, Tasmania comprises 1,092 private residences [14]. Throughout the initiation phase, a total of 899 (82%) residences were visited, of which 46% were confirmed to have wood heaters. There was also identification of two open fires and one wood pellet burner. Of the residences with wood heaters, 283 (approximately 68%) received a catalytic device during door-to-door distribution on June 14 and 15, 2014. The remaining residents either declined the offer of a catalyst, or were not at home and a flyer explaining the study and how to get a free catalyst was left at their door. By the end of July 2014, a further 43 catalysts had been collected from the Perth Roadhouse and the proportion of wood heaters with a catalyst reached 78%. A small proportion of residences (less than 1%) did not have a mailbox or visitor access and as a consequence were not offered a catalyst.

### Ambient PM_2.5_ in all communities

#### Descriptive Statistics

**Fig 3:**
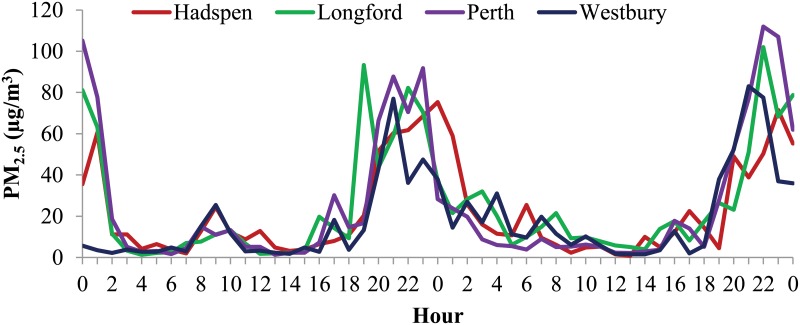
Hourly ambient PM_2.5_ concentration over two consecutive smoky days (June 29 and 30, 2014), showing the similar diurnal pattern of pollution between the four towns.

All four sampled towns recorded similar mean particulate concentrations between pre- and post- intervention phases (Table 1). Both the mean PM_2.5_ concentrations and the number of days in which the 24 hour average PM_2.5_ exceeded 25μg/m3 tended to increase, post- intervention, for all locations. The highest concentrations were recorded for Longford (Table 1). Fig 3 demonstrates the general correlation in air quality between the towns, with two consecutive days of mean hourly PM2.5 concentrations which tended to fall to a minimum around midday and peak during the early morning, evening and around midnight in all locations.

**Table 1:** Mean PM_2.5_ concentration and meteorological parameters, temperature, wind speed, relative humidity and atmospheric pressure, by location and intervention phase.

Location	Perth [intervention]		Longford		Hadspen		Westbury	
Intervention Phase	Pre	Post	Pre	Post	Pre	Post	Pre	Post
**Total Days**	160	90	160	90	160	90	160	90
**Mean PM2.5 [μg/m**^**3**^**]**	17	20	19	22	17	19	12	15
**Days above 25μg/m**^**3**^	41(26)	26(29)	48(30)	33(37)	42(26)	27(30)	7(4)	14(16)
**Mean Temperature (°C)**	9.5	9.1	8.8	8.4	9.2	8.7	8.3	8
**Mean Wind Speed (m/s)**	1	0.9	0.4	0.4	1	1	0.7	0.6
**Mean Relative Humidity (%)**	84	82	85	84	85	84	86	84
**Mean Atmospheric Pressure (hPa)**	1017	1020	1015	1018	1016	1019	1016	1019

#### Statistical analyses

Simple comparisons of PM_2.5_ concentrations before and after the intervention and the ratio of average PM_2.5_ concentrations between Perth and the three control towns showed little change before and after the intervention. Sophisticated statistical modelling was conducted to evaluate changes in air quality after adjusting for meteorological and day of week effects. This was implemented in two stages.

**Fig 4:**
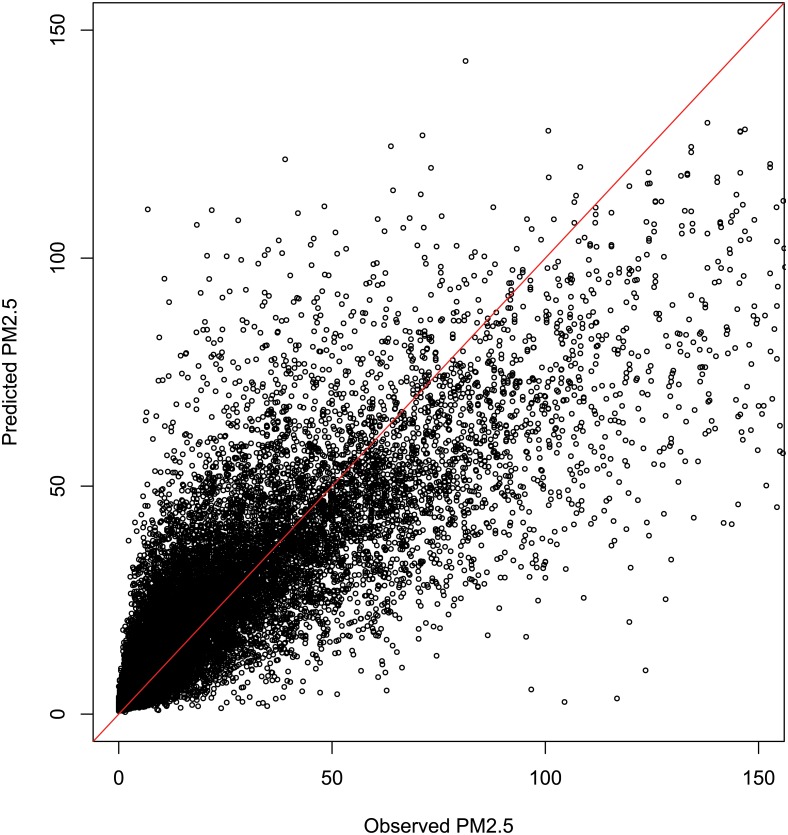
Observed versus predicted PM_2.5_ RandomForest model output. The model had a RMSE for the response variable PM_2.5_ of 13.6 μg/m^3^.

In stage one, we modelled the influence of meteorological conditions and day of week on daily PM concentrations. The RandomForest model performed well in producing residual PM_2.5_ values adjusted for meteorological and temporal patterns, with a root mean square error (RMSE) of 13.6μg/m^3^ and a close to linear response. Observed and predicted values of PM_2.5_ from the RandomForest model are shown in Fig 4.

**Fig 5:**
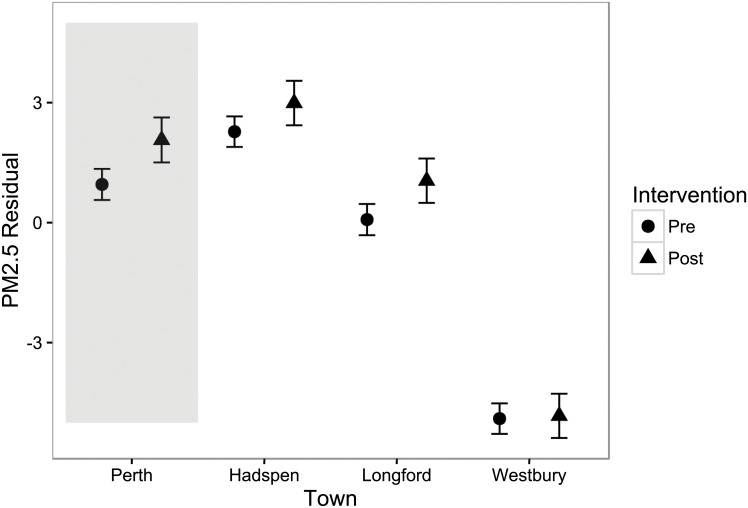
Interaction plot with 95% confidence intervals of GLM model for intervention and town. While there were significant differences between towns in PM_2.5_ residuals, there were no significant declines in PM_2.5_ during the intervention period, including in the town with Smartburn units installed, Perth.

The outputs of the second stage of the statistical analyses are shown in Table 2. While individual towns had different PM_2.5_ values from each other, regardless of the intervention period, there were no statistically significant changes to the ambient air quality between pre- and post-intervention phases in any of the towns. Indeed in most cases there were slight increases in ambient PM_2.5_ (Fig 5). The hypothesis that the firebox catalyst intervention would result in a reduced average PM_2.5_ concentration in Perth was therefore not supported. Equivalent models performed for specific times of 7.00am, 6.00pm and 11.00pm which is when daily peaks in PM typically occurred, also showed no significant interaction effect (p>0.05).

**Table 2:** Intervention by town GLM output table, showing significant differences (**) between towns in PM_2.5_ residuals, but no significant effect of the intervention period, nor a significant intervention effect in any individual town. Values Relative to Hadspen (Intercept).

Coefficient	Estimate	Std. Error	t value	p value
**Intercept**	2.32	0.19	-11.91	<0.01 **
**Intervention**	0.66	0.34	-1.92	0.55
**Longford**	-2.22	0.28	7.96	<0.01 **
**Perth**	-1.39	0.28	5.00	<0.01 **
**Westbury**	-7.25	0.28	26.33	<0.01 **
**Intervention:Longford**	0.32	0.49	-0.66	0.61
**Intervention:Perth**	0.45	0.49	-0.91	0.36
**Intervention:Westbury**	-0.53	0.49	1.11	0.27

### Participant survey

Approximately 78% of the Perth sample population provided consent for contact, via email or telephone and 131 (52%), of these responded to the post intervention survey, 100 by telephone and 31 by email. The results are presented in Table 3. As recommended by the Tasmanian EPA [18], almost half of respondents reported that they allowed twenty minutes before reducing primary air intake after ignition and reloading. Firewood was stored in a dry, sheltered area by most (70%) respondents for up to six months (54%). About half reported that they maintained the canister elevated and away from direct heat for at least two months (91%). About 40% reported clearance of soot from the flue or glass door, reduced smoke production from the firebox, or thought that their fires appeared to burn hotter or longer, all of which are consistent with correct function of the catalyst. Almost half stated that they intended to purchase the device in the future.

**Table 3:** Responses from 131 participants surveyed three months following the intervention.

**Age of wood heater (years)**			**Canister location in firebox**^**2**^		
**< 10**	52	40%	**Elevated**	9	7%
**10 to 20**	45	34%	**Side**	119	91%
**> 20**	22	17%	**Neither**	12	9%
**Unsure**	12	9%	**Unsure**	0	
**Duration air inlet fully open after reloading (minutes)**			**Canister duration (months) in firebox**		
**> 20**	61	47%	**2 or less**	11	9%
**10 to 20**	34	26%	**More than 2**	119	91%
**5 to 10**	13	10%			
**< 5**	11	8%			
**Unsure**	12	9%			
**Duration air inlet fully open after ignition (minutes)**			**Heater observations during canister use**^**2**^		
**> 20**	65	50%	**No change**	67	51%
**10 to 20**	47	36%	**Cleaner glass**	40	31%
**5 to 10**	12	9%	**Cleaner flue**	12	9%
**< 5**	3	2%	**Longer burn**	11	8%
**Unsure**	4	3%	**Less smoke**	15	11%
**Conditions of firewood storage**			**Hotter fire**	11	8%
**Dry and sheltered, inside**	73	85%	**Unsure**	10	8%
**Outside and unsheltered**	19	15%			
**Duration of firewood storage**					
**Up to 6 months**	71	54%			
**6–12 months**	0	0			
**1–2 years**	35	27%			
**> 3 years**	8	6%			
**Unsure**	17	13%			

## Discussion

Contrary to our prediction, community-wide distribution of the catalytic device was not associated with a significant change in the ambient winter air quality of Perth. Possible explanations for this finding may include: [1] inadequate or biased participation of households in the intervention, [2] non-representative sampling of ambient air quality, for example any change in pollution may have been too small for our monitoring techniques to detect, [3] poor operation of the catalyst under field conditions, for example due to inadequate maintenance of the catalyst and poor combustion conditions in highly polluting heaters, [4] differences in meteorological conditions before and after the intervention, or [5] improvements associated with use of the catalyst were not large enough to be detected within the constraints of the statistical model used. These are discussed further below.

Although 78% of houses with wood heaters accepted a catalyst, in many cases we were not able to directly confirm that it was actually installed. However, the study was met with considerable community enthusiasm and support and we think that the vast majority of distributed catalysts were likely to have been inserted in the fireboxes. However, it is possible that the people who accepted the firebox device were more likely to be interested in the operation of their heaters, and less likely to be the major contributors to poor ambient air quality than the group of people who did not accept the device. Generation of particulate air pollution is highly variable between individual wood heaters and dependent upon operation of the heater. Within a community, only a relatively small proportion of households tend to produce the majority of particulate emissions [19]. If the 20% of households missed by the intervention included a high proportion of households in this high emissions group, it is possible that even large reduction in emission from heaters that are already burning efficiently, will not be measurable if these were not the main source of community wide air pollution. Close proximity to the air monitoring station of relatively smoky chimneys is another factor that may have reduced our ability to detect any reductions in emissions achieved throughout the wider community.

Maintenance of the catalyst within the firebox may have been inadequate. The participant survey highlighted that more than half of the respondents failed to maintain the canister elevated out of the ash, potentially disabling the action of the device. Many participants had no means of preventing burial of the device in the ash-bed, which blocks the release of the active constituents of the product into the firebox. Consistent with this was the observation that more than half the respondents did not observe any signs of catalyst-related changes to their wood heater such as cleaner glass (Table 3). Future development of product design may be useful in addressing this, especially as 47% of respondents intended to purchase the device again. Design features, such as a simple folding stand could overcome the difficulty of maintaining level and elevated placement of the canister, away from excessive heat within the firebox.

Poor firebox conditions for efficient combustion among some participating households may have also inhibited catalyst function. Use of wood with a high moisture content, insufficient provision of air and placement of logs in configurations that restrict circulation of oxygen within the firebox create conditions of low-temperature smouldering combustion, under which catalytic activation is inhibited [19,20]. Catalytic performance might also be inhibited by durations of hot, rapid combustion, under which sustained airflow and elevated temperature increase the rate of vaporisation and exhaustion of the catalytic mixture [19]. Complete vaporisation of the catalyst may have occurred in less than three months, although no significant improvement was observed early in the study period. Almost all respondents reported that they allowed maximum combustion air for at least 20 minutes before reducing air intake after starting or re-loading their heaters, and used firewood that had been stored in a dry, sheltered area for six months before burning (Table 3) However, self-reported information about wood burning practices could well be unreliable. Variability in these wood moisture and combustion conditions would have influenced combustion temperature and subsequent activation and function of the catalyst [20].

In laboratory trials, installation of the catalyst is associated with reduced particulate emissions from individual wood heaters [13]. However, these findings are reported through commercial sources and there is little information about performance of catalysts in field studies, with operation of wood heaters under ‘real- world’ conditions. The only other study to have trialled the device in a community setting concluded that use of the catalyst was associated with reduced emissions from individual chimneys [9]. However differences in experimental design are important to note. Hine et al. [9] visually assessed smoke plume density generated from individual wood heaters, while in our study we monitored the overall particle concentration in ambient air.

While meteorological conditions have a large influence on ambient air quality, we do not think that this influenced the results of our study. Consistent with previous studies we found that calm, humid conditions and higher atmospheric pressure were all associated with elevated PM_2.5_ concentrations [4,21]. This may be because high pressure systems create calm, clear conditions that are conducive to the formation of thermal inversions [4,21]. Meteorological conditions were generally similar between the four towns and their influence was taken into account in the statistical analysis. However, we were unable to consider all significant influential factors upon PM concentration, such as atmospheric vertical mixing and the presence of inversion layers, due to the limited data availability for the region.

Finally, it is also possible that there were subtle changes in air quality not detectable in the context of the background ‘noise’ in the data. When we compared our modelled and empirical PM_2.5_ values after adjustment for meteorological and temporal patterns, we found a linear relationship with a RMSE of 13.6μg/m^3^. Any improvement attributable to the intervention of a lesser magnitude would be unlikely to be detected.

Strengths of this study included the success of community awareness-raising strategies, with a large sample population encompassing almost 80% of Perth households with wood heaters. Continuous ambient air sampling at 10-minute intervals from BLANkET stations across four locations was almost complete with only a few hours of data missing due to temporary malfunction. This allowed us to conduct detailed analysis and adjust for the roles of meteorological conditions in each individual community. Limitations of the study included the subjective evaluation of maintenance of the catalyst and wood heater operation in individual households. Our results suggest that these factors could be extremely important in achieving the benefits of catalysed combustion and should be investigated in detail in future studies.

In rural Tasmania, and similar settings throughout southern Australia, ambient winter pollution from wood heaters remains an important public health problem. Affordable and accessible strategies to reduce pollution are needed. Firebox catalysts have the potential to play a role but further research is required. An economical evaluation of community-wide catalyst installation may be conducted in comparison with other interventions, adopting the approach of wood heater change-out. Future interventions may devise strategies to engage all residents of a community, especially those of the most polluting wood heater households and objectively measure and evaluate the influence of placement of the catalyst, the use of dry wood and operation of the heater upon ambient emissions. For example, the use of temperature loggers on the flue is a non-invasive objective way to provide information about the state of combustion and the rate of air infiltration into the firebox. Public interest in the catalytic device that was found to exist within the community provides incentive for future research and development of the product to further evaluate its potential to influence air quality.
